# Immune features and clinical characteristics of chikungunya fever in children: differences from adults and across pediatric age groups

**DOI:** 10.3389/fped.2026.1863169

**Published:** 2026-08-25

**Authors:** Yilin Li, Shunhang Wen, Hailin Zhang

**Affiliations:** Department of Pediatric Respiratory Medicine, The Second Affiliated Hospital and Yuying Children’s Hospital of Wenzhou Medical University, Wenzhou, China

**Keywords:** arbovirus, chikungunya fever, child, disease attributes, immunity

## Abstract

Chikungunya fever (CHIKF) is an acute infectious disease transmitted by *Aedes* mosquitoes, with primary symptoms including fever, rash and joint pain. The immune features and clinical manifestations of CHIKF in children differ from those observed in adults. Following chikungunya virus (CHIKV) infection, children more frequently present with high fever, rash, and neurological complications compared with adults, whereas arthralgia and chronic manifestations are less commonly observed. Clinical features also vary among pediatric age groups. This review summarizes current evidence on the age-related immunological and clinical characteristics of CHIKV infection and highlights their implications for pediatric disease management.

## 1 Introduction

**Figure 1:**
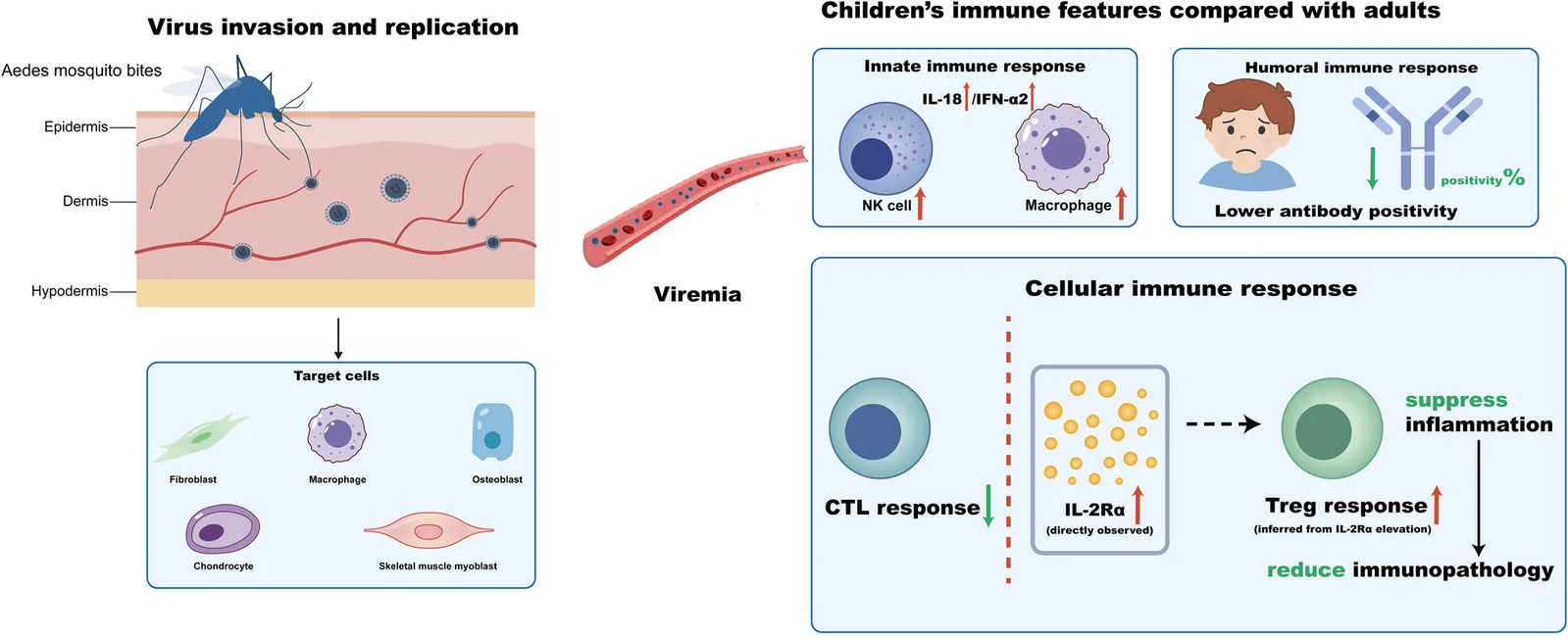
Schematic overview of CHIKV invasion, viremia, and pediatric immune features in CHIKF. CHIKV is introduced into the skin by Aedes mosquito bites, infects target cells including fibroblasts, macrophages, osteoblasts, chondrocytes, and skeletal muscle myoblasts, then disseminates through the bloodstream to cause viremia. Compared with adults, children exhibit enhanced innate immune features, characterized by increased IL-18 and IFN-α2 levels and greater activation of NK cells and macrophages, together with lower antibody positivity and a weaker CTL response. Increased IL-2Rα expression is directly observed and is suggestive of an enhanced Treg response, which may suppress excessive inflammation and thereby reduce immunopathological damage. Red upward arrows and green downward arrows indicate relative increases/activation and decreases, respectively. Abbreviations: CHIKV, chikungunya virus; CHIKF, chikungunya fever; IL-18, interleukin-18; IFN-α2, interferon-α2; NK, natural killer; CTL, cytotoxic T lymphocyte; IL-2Rα, interleukin-2 receptor alpha chain; Treg, regulatory T cell.

Chikungunya fever (CHIKF) is an acute infectious disease caused by chikungunya virus (CHIKV), primarily transmitted through the bites of Aedes mosquitoes. Its typical clinical manifestations include fever, rash, and severe joint pain (1, 2). Vertical mother-to-child transmission primarily occurs during intrapartum maternal viremia, often causing severe symptomatic neonatal infection (3). The immune characteristics and clinical presentation of pediatric CHIKF differ from those in adults (Figure 1), particularly in neonates and children with compromised immune systems, who may develop severe complications following CHIKV infection. Some children may experience long-term sequelae, including epilepsy, developmental delays, and movement disorders (4). This review summarizes current evidence on the immune features and clinical characteristics of CHIKF in children, with emphasis on differences between children and adults and variation across pediatric age groups.

## 2 Virus invasion and replication

After transmission by infected mosquitoes, CHIKV initially replicates in skin and subcutaneous tissues. Its target cells include fibroblasts, macrophages, osteoblasts, chondrocytes, and skeletal muscle myoblasts (1). After rapid replication in local tissue, the virus spreads throughout the body via the bloodstream, leading to viremia (5). Matrix remodeling-associated protein 8 (MXRA8) is a specific receptor for CHIKV that facilitates viral attachment and entry into cells; it is highly expressed in cells such as synovial fibroblasts, osteoblasts, and chondrocytes, and is considered one mechanism underlying arthralgia in patients with CHIKF (6, 7).

Pediatric susceptibility is likely influenced not only by virological factors but also by exposure patterns, skin and barrier physiology, developmental immunity, and comorbid conditions. Children have weaker mosquito protection awareness, making them more susceptible to mosquito bites. Additionally, their thinner skin increases the risk of viral invasion (6, 7). Neonates typically exhibit higher post-infection viral loads than older children, likely reflecting neonatal immune immaturity (8).

## 3 Immune features

### 3.1 Innate immune response

Innate immunity constitutes the first line of host defense against viral infection and is rapidly engaged during CHIKV infection. CHIKV infection markedly activates circulating monocytes, particularly CD16 + subsets, and increases pro-inflammatory cytokines and chemokines production (9, 10). Compared with adults, children may initiate a more rapid innate immune response after CHIKV infection. Early increases in IL-18 and IFN-α2 may enhance NK-cell and macrophage activation, thereby promoting early viral clearance (11).

### 3.2 Humoral immune response

Compared with adults, children have a lower antibody positivity rate after CHIKV infection. In a study in Nicaragua (12), the antibody positivity rates after infection were 6.1% for the 2–14-year-old group and 13.1% for individuals aged 14 and above. However, antibody persistence remains comparable between children and adults, with most pediatric patients establishing long-lasting humoral immunity (13).

### 3.3 Cellular immune response

Cytotoxic T lymphocytes (CTLs) and regulatory T cells (Tregs) play critical roles in reducing chronic CHIKV infection. CTLs are important for eliminating infected cells, whereas regulatory Tregs restrain excessive inflammation and limit immune-mediated tissue injury (14, 15). Although attenuated CTL activity in children could theoretically favor viral persistence, chronic arthralgia and other post-acute manifestations are reported less frequently in pediatric patients than in adults (16). This discrepancy suggests chronicity depends on the balance between early antiviral control and inflammatory regulation rather than CTL strength alone. Furthermore, higher IL-2R*α* levels have been observed in children following CHIKV infection (11), suggesting a stronger Treg-related regulatory response that may help reduce inflammation and joint tissue damage. However, because Tregs were not directly assessed, elevated IL-2R*α* alone cannot confirm an actual increase in Treg numbers or activity.

### 3.4 Lymph node immune memory

Lymph nodes are essential organs of the immune response, playing a pivotal role in the initiation and early development of adaptive immune memory. In murine models, CHIKV damages local lymph nodes by recognizing and binding to the macrophage receptor with collagenous structure (MARCO), a scavenger receptor expressed on the surface of lymphatic endothelial cells (LECs). This binding mediates viral entry and destruction of LEC architecture. This process can trigger local inflammation and continuously impair the antigen-capturing function of LECs, which is crucial for establishing long-term immune memory (17). This finding is particularly relevant for children, whose maturing immune systems require continuous antigen exposure to establish immunological memory. CHIKV infection can disrupt the function of LECs, hindering the formation of diverse immunological memory in children and affecting the response to subsequent infections or vaccinations.

## 4 Clinical characteristics

### 4.1 Typical symptoms

#### 4.1.1 Fever

The primary symptom of CHIKF is fever. A higher proportion of children develop high fever compared with adults, with body temperatures often exceeding 39 °C, typically lasting 3–5 days, and following an irregular pattern. The prevalence of high fever appears to increase with decreasing age, and younger children may also experience a longer duration of fever (18). Tavares et al. (19) reported that fever duration in children with CHIKF ranged from 2 to 19 days due to individual differences, with a median duration of four days. Animal studies demonstrate that mice reduce energy production after CHIKV infection to inhibit viral replication, whereas rhesus macaques show enhanced metabolism to provide energy for a more robust immune response (20). We propose early pediatric metabolic regulation resembles that of rhesus macaques: enhanced metabolic activity drives cytokine production and elevates pyrogenic mediators, such as IFN-α2, possibly raising the hypothalamic thermoregulatory set point (11). Together with the intrinsically higher basal metabolic rate in children, this mechanism may partly explain their greater susceptibility to high-grade fever. Nevertheless, this mechanistic hypothesis requires further validation through direct metabolomic and immunological investigations in pediatric patients with CHIKF.

#### 4.1.2 Rash

Rashes are more common in children than in adults, and they appear earlier. Whereas in adults rashes typically appear 4–6 days after fever onset, in children they often appear on the first day of illness, usually as maculopapular eruptions commonly distributed across the body (21, 22). Furthermore, children are more prone to hyperpigmentation, which can appear rapidly within a few days of fever and rash onset. It is characterized by brown hyperpigmentation at the tip of the nose, referred to as “Chik sign” or “Brownie nose sign,” often accompanied by hyperpigmentation on the auricle (22). The higher incidence of rashes in children may be related to factors such as thinner skin, more active Langerhans cells in the epidermis, and a stronger inflammatory response (23).

#### 4.1.3 Arthralgia

The incidence, recurrence rate, and risk of chronicity of joint symptoms in children are lower than in adults (16). A prospective cohort study of 770 participants with chikungunya in Managua, Nicaragua, demonstrated an age–dependent increase in the prevalence of arthralgia. The proportion of participants reporting acute arthralgia increased from 77.5% among children aged 0–4 years to 98.4% among those aged ≥16 years. Across the pediatric age groups, 18.6%–32.5% of participants reported arthralgia at two or more study visits, compared with 55.1% of adults. Among children, post–acute polyarthralgia was most prevalent in the legs, followed by the hands and torso/elbows, whereas no significant differences in its distribution among body regions were observed in adults (24). Retrospective data from 343 children with CHIKF in Foshan supported these findings, showing that 52.19% of children had ankle arthralgia and 31.78% had wrist arthralgia. None of the children experienced arthralgia at discharge or significant joint discomforts one week post-discharge (25).

A meta-analysis by Teng TS et al. (26) revealed a close association between elevated pro-inflammatory cytokines and the occurrence of arthritis in patients with acute CHIKV infection. IL-17 is a key cytokine that induces severe arthralgia. Experimental modes demonstrate that during the acute phase of infection, IL-17 suppresses IFN-α2 expression, creating favorable conditions for viral replication, and promotes early inflammatory cell infiltration, leading to acute joint swelling and fever. In the chronic phase, IL-17 induces the expression of matrix metalloproteinases (MMPs), promoting extracellular matrix degradation, which damages cartilage structure and impairs tissue repair capacity. In parallel, IL-17 activates the NF-*κ*B pathway, promoting the expression of pro-inflammatory factors such as TNF-α, IL-1β, and IL-6, exacerbating tissue inflammation and ultimately leading to chronic joint damage (27–29). Teixeira et al. (30) further indicated that the involvement of IL-17 in the immune network of children is less prominent than that in adults. Children infected with CHIKV show elevated levels of monocyte chemotactic factors and activation of Th2-type immune responses, which may explain the milder joint symptoms observed in children.

Granulocyte-macrophage colony-stimulating factor (GM-CSF) is typically a key pro-inflammatory mediator involved in arthritis pathogenesis. However, Simarmata et al. (11) found that children with low GM-CSF expression following infection were more likely to experience arthralgia. This apparent paradox may arise because high GM-CSF levels facilitate early viral clearance through pro-inflammatory mechanisms, thereby indirectly mitigating subsequent joint damage.

### 4.2 Complications

#### 4.2.1 Nervous system complications

CHIKV infection affecting the nervous system can manifest as meningitis, seizures, myelitis, and Guillain-Barré syndrome, among other disorders. Among these, meningitis is the most common, typically occurring shortly after systemic symptoms such as fever and rash, and presenting with severe headache, neck stiffness, and altered consciousness. Seizures occur more frequently in children than in adults, and younger children are more prone to status epilepticus. Myelitis is relatively rare; affected patients experience limb weakness and sensory loss associated with direct viral invasion of neural tissues. CHIKV-associated Guillain-Barré syndrome has a 3–17-day prodrome, presenting with symmetric bilateral flaccid weakness, sensory abnormalities, and cranial nerve paralysis. Most patients have a favorable prognosis and recover within three months (31, 32).

#### 4.2.2 Other complications

Apart from neurological complications, non-neurological systemic complications are uncommon in most pediatric CHIKF series but remain clinically important when present. Cardiovascular involvement can include myocarditis, ventricular dysfunction, arrhythmia, and, rarely, death (33). Ocular complications include conjunctivitis, uveitis, optic neuritis, ocular pain, and decreased visual acuity; however, published data primarily derive from adult or mixed-age cohorts rather than pediatric-specific populations (34). The relative scarcity of pediatric reports may occur because ocular symptoms are less common in children, or because young children cannot accurately articulate vision disturbances, making mild ocular lesions more likely to be overlooked.

Hepatic and renal dysfunction are usually reported in severe disease or in patients with additional risk factors. Some children with liver damage may progress to liver failure and may require liver transplantation (35). Acute kidney injury most commonly manifests as acute interstitial nephritis, characterized by oliguria and elevated serum creatinine levels. The pathogenesis may involve direct viral damage to renal tubules and shock leading to renal hypoperfusion. Mercado et al. (36) reported in a study of fatal CHIKF cases that acute kidney injury is more common among older adults and infants, possibly due to weaker immune resistance in these groups, with poor renal recovery in older adults and immature renal development in infants.

### 4.3 Characteristics of CHIKF in children of different age groups

In this review, neonates are defined as patients aged ≤ 28 days, infants as aged 29 days to 1 year, and children as aged > 1 year. Across these three age groups, pediatric patients infected with CHIKV exhibit distinct clinical characteristics.

#### 4.3.1 Neonates

Neonatal infection is primarily associated with vertical transmission, and transmission risk is strongly determined by the timing of maternal infection. When maternal infection occurs near delivery, the transmission risk can reach 50%, whereas infection occurring seven days before delivery almost never results in vertical transmission. Affected neonates typically develop symptoms 3–7 days after birth, including fever, rash, limb edema, and neurological involvement (3). Although maternally derived CHIKV-specific IgG may be transferred to neonates, this passive protection may be insufficient due to neonatal immune system immaturity and incomplete blood–brain barrier development; this partly explains why neonates can develop higher post-infection viral loads, more severe disease, and more extensive multi-organ involvement than older children (8). Neurological manifestations are particularly prominent in this age group and may progress to meningitis, brain edema, intracranial hemorrhage, or acute necrotizing encephalopathy. Some neonates also develop sepsis-like presentations, including circulatory failure and coagulation dysfunction; severe cases may progress rapidly to multi-organ failure (32), suggesting that neonatal disease is not merely an early-age presentation of pediatric CHIKF, but a distinct clinical entity shaped by perinatal exposure, immature barrier function, and developing antiviral immunity.

The long-term consequences of neonatal CHIKV infection are a major concern. A cohort study conducted on Reunion Island reported that approximately 50% of children with perinatal mother-to-child CHIKV infection had global neurodevelopmental delay at around two years of age (37). Ferreira et al. (38) showed that neonates exposed to CHIKV, even in the absence of clinically apparent infection, had a significantly higher incidence of neurodevelopmental abnormalities, indicating that symptomatic infection alone may underestimate the developmental burden of perinatal CHIKV exposure. Pregnant women with confirmed CHIKV infection should therefore undergo ultrasound assessment every four weeks to monitor fetal growth, development, and amniotic fluid status, with the interval shortened to every two weeks near term or when complications are suspected. Neonates exposed to CHIKV *in utero* require careful assessment for acute neurological signs and long-term neurodevelopmental outcomes (39). In addition to neurological disease, neonatal CHIKV infection may involve the cardiovascular system, most commonly presenting as coronary artery dilation and myocardial hypertrophy, but also as ventricular dysfunction or pericarditis (40). However, the mechanisms, incidence, and long-term prognosis of cardiovascular involvement remain insufficiently defined. No antiviral therapy or immunotherapy has been established for neonatal CHIKV infection; immunoglobulin therapy remains experimental and requires further clinical validation (39).

#### 4.3.2 Infants

In infants, CHIKV infection is most often recognized by fever, rash, and irritability, whereas joint involvement may be underestimated because infants cannot reliably express pain. Atypical rashes are more common in infants. In a study of 1,469 patients with confirmed CHIK infection in Puerto Rico, vesicular eruptions were identified in 9.8% of the 41 hospitalized infants; importantly, all affected infants were classified as severe cases, and no vesicular rashes were reported in other age groups (41). Infants are more likely than older children to develop severe disease and neurological complications (16, 41); the mechanisms underlying this age-related vulnerability may include immature skin, mucosal, and blood–brain barriers, as well as developing innate and adaptive immunity. Mesquita Ramirez et al. (42) reported that infants with CHIKV infection predominantly presented with high fever, rash, and circulatory compromise, whereas arthralgia and seizures were relatively uncommon. Compared with older children, infants were at a higher risk of developing septic shock, and age ≤ 60 days was identified as an independent risk factor, underscoring the need for careful hemodynamic assessment in infants with suspected CHIKV infection during outbreaks to enable early identification and prompt management of septic shock. In addition to acute circulatory complications, the potential long-term neurological consequences of CHIKV infection in infancy warrant particular attention.

#### 4.3.3 Children

In children (> 1 year), the clinical manifestations of CHIKV infection are generally similar to those observed in adults, with fever, rash, and arthralgia as the predominant symptoms. Overall, disease severity in this group is relatively mild, and recovery during the acute phase is usually rapid. With increasing age, the risk of neurological complications declines, whereas the incidences of rash, arthralgia, and chronic manifestations tend to rise (13, 16, 31).

## 5 Asymptomatic infection

In children, particularly younger children, infection symptoms are often overlooked due to limited expression abilities, passive healthcare-seeking behavior, and atypical clinical presentation. Approximately 40% of pediatric CHIKV infections have been estimated to be asymptomatic (43). However, de Jesus Pereira et al. (13) recently challenged this estimate using an active surveillance strategy, reporting that 90.6% of infected children developed symptoms. This marked discrepancy may partly reflect differences in surveillance intensity, case ascertainment, population characteristics, and follow-up duration. Passive or cross-sectional approaches may fail to capture mild or transient symptoms, whereas active surveillance increases the likelihood of symptom detection. Findings from a single actively monitored cohort may not represent all outbreak settings or pediatric populations. The true proportion of asymptomatic CHIKV infection in children remains unclear.

## 6 Vaccine

Currently, CHIKV vaccines can be broadly classified into five categories: inactivated, live-attenuated, subunit, virus-like particle (VLP), and recombinant viral vector vaccines (44). To date, two CHIKV vaccines have been approved for use in the United States. The application of the live-attenuated vaccine IXCHIQ remains limited due to safety concerns, with several cases of serious adverse events reportedly associated with vaccination (45). The VLP-based vaccine VIMKUNYA demonstrated a favorable safety profile and has been approved for use in individuals aged 12 years and older (44). Inactivated and recombinant viral vector vaccines have shown promising safety and efficacy profiles in trials (46–48). Subunit vaccines induce neutralizing antibody responses in animal studies, but their ability to provide broad cross-protection against different CHIKV lineages remains limited (49).

## 7 Future directions and research gaps

Current evidence on pediatric CHIKV infection remains limited, particularly regarding age-specific immune responses and their changes over time. Prospective longitudinal studies are needed to characterize viral kinetics, innate and adaptive immune responses, and clinical outcomes across different pediatric age groups. Pediatric vaccine data, especially in younger children, remain insufficient; further studies should assess age-specific safety, immunogenicity, and durability of protection. Although neurodevelopmental abnormalities have been reported after intrauterine or perinatal CHIKV exposure, their long-term course and associated factors remain unclear. Standardized, age-appropriate neurodevelopmental assessments and extended follow-up are therefore required.

Neurological manifestations may also vary among CHIKV genotypes, although findings remain inconsistent across experimental systems. Clinical observations suggest that the ECSA genotype may be more frequently associated with neurological manifestations (50). In 4–6-week-old mice, ECSA infection induced stronger pro-inflammatory responses and greater CD4⁺ T-cell infiltration, whereas in human monocyte-derived macrophages it showed higher replication titers but lower cytokine production than the Asian genotype (51, 52). In contrast, suckling-mouse experiments reported higher mortality following Asian-genotype infection, possibly associated with earlier intracerebral apoptosis (53). These discrepancies indicate that genotype-related neurovirulence is influenced by host age, immune status, target cell type, and infection stage. Future studies using human-derived or physiologically relevant models are needed to clarify how viral replication, host immune responses, cell-death pathways, and genotype-specific viral factors contribute to neurological injury.

## 8 Summary

Pediatric CHIKV infection differs from adult infection in both immune regulation and clinical expression. Current evidence suggests that children may achieve effective control of acute infection while experiencing less persistent joint disease, although the cellular and molecular mechanisms underlying these age-related differences remain unclear. Clinically, pediatric CHIKV infection is characterized by frequent fever and rash, generally milder joint involvement, and a greater risk of neurological complications, with substantial variation across pediatric age groups. Recognition of these age-related differences is essential for pediatric risk assessment, clinical monitoring, and follow-up.
